# Plant Ecological Strategies Shift Across the Cretaceous–Paleogene Boundary

**DOI:** 10.1371/journal.pbio.1001949

**Published:** 2014-09-16

**Authors:** Benjamin Blonder, Dana L. Royer, Kirk R. Johnson, Ian Miller, Brian J. Enquist

**Affiliations:** 1 Department of Ecology and Evolutionary Biology, University of Arizona, Tucson, Arizona, United States of America; 2 Rocky Mountain Biological Laboratory, Gothic, Colorado, United States of America; 3 Department of Earth and Environmental Sciences, Wesleyan University, Middletown, Connecticut, United States of America; 4 National Museum of Natural History, Smithsonian Institution, Washington, DC, United States of America; 5 Denver Museum of Nature and Science, Denver, Colorado, United States of America; 6 The Santa Fe Institute, Santa Fe, New Mexico, United States of America; University of California, Berkeley, United States of America

## Abstract

The Chicxulub bolide impact caused the end-Cretaceous mass extinction of plants, but the associated selectivity and ecological effects are poorly known. Using a unique set of North Dakota leaf fossil assemblages spanning 2.2 Myr across the event, we show among angiosperms a reduction of ecological strategies and selection for fast-growth strategies consistent with a hypothesized recovery from an impact winter. Leaf mass per area (carbon investment) decreased in both mean and variance, while vein density (carbon assimilation rate) increased in mean, consistent with a shift towards “fast” growth strategies. Plant extinction from the bolide impact resulted in a shift in functional trait space that likely had broad consequences for ecosystem functioning.

## Abstract 2

The End-Cretaceous Impact Winter Killed Off Slow-Growing Plants The end-Cretaceous mass extinction caused the selective extinction of plant species with slow-growth strategies, consistent with an impact winter hypothesis.

## Author Summary

Sixty-six million years ago the Chicxulub bolide impacted the Earth, marking the Cretaceous–Paleogene boundary (KPB). This event caused the planet's most recent mass extinction, but the selectivity and functional consequences of the extinction on terrestrial plants has been largely unknown. A key untested hypothesis has been that a subsequent impact winter would have selected against slow-growing evergreen species, a possible cause of the modern dominance of high-productivity deciduous angiosperm forests. We tested this hypothesis using fossil leaf assemblages across a 2-million-year interval spanning the KPB. We assess two key ecological strategy axes—carbon assimilation rate and carbon investment—using leaf minor vein density and leaf mass per area as proxies, respectively. We show that species that survive the KPB have fast-growth ecological strategies corresponding to high assimilation rates and low carbon investment. This finding is consistent with impact winter leading to the nonrandom loss of slow-growing evergreen species. Our study reveals a dramatic example of the effect of rapid catastrophic environmental change on biodiversity.

## Introduction

The Cretaceous–Paleogene boundary (KPB) is marked by the Chicxulub bolide impact and mass extinction [1]–[3]. In temperate North America, while the impact resulted in the extinction of more than 50% of plant species [4], a major unresolved issue is whether this killing event was also a large-scale selection event [5]. Wolfe [6] originally proposed that the KPB selected against evergreen species. Specifically, competition in the cold and dark climates during the impact winter [7] should have selected for species with ecological strategies [8] associated with deciduousness. Because variation in leaf traits reflect ecological strategies that are coupled to whole-plant carbon and water fluxes, such a strategy shift would also have caused broad shifts in terrestrial ecosystem functioning [9],[10]. Although some fossil data are consistent with the rise of deciduous species near the KPB, inferences on the extinction's selectivity have been based on qualitative proxies or limited occurrence data [11]–[14]. A lack of quantitative trait data has limited our understanding about the selectivity and ecological implications of this important extinction event.

Here we test if the extinction event was selective for functional traits related to plant ecological strategies. We measure functional traits from fossil leaf assemblages spanning a 2.2 Myr interval across the KPB and assess four differing selection scenarios for functional traits: (i) directional selection—a shift in phenotype space, caused by novel postboundary environments replacing preboundary environments; (ii) stabilizing selection—a reduction in phenotype space caused by postboundary conditions making some strategies temporarily nonviable; (iii) diversifying selection—an increase in phenotype space due to a wider range of postboundary conditions; or (iv) a lack of selection, because previous adaptations were unrelated to survival after major catastrophe.

We assess evidence for these selection scenarios using functional traits [15],[16] related to the leaf economics spectrum [17]. This global spectrum details how several traits are linked to variation in plant growth and fitness. It describes a continuum between “slow-return” leaves (low rates of carbon assimilation, long lifespans, and high tissue carbon investment) and “fast-return” leaves (high rates of carbon assimilation, short lifespans, and low tissue carbon investment). In general, evergreen leaves are “slow.” Within angiosperms, this strategy is thought to be selected for when resource availability is less variable [18]. In contrast, “fast” deciduous angiosperm leaves are thought to be selected for when resource availability is more variable [19]. In this updated context, Wolfe's hypothesis [6] proposed that the strong variation in light levels and temperature after the bolide-caused impact winter [1] should have resulted in directional selection for “fast” strategies.

Alternatively, longer-term temperature change may also have selected for certain traits across the KPB. Multiple proxies show a brief warming during the latest Cretaceous, starting approximately 300,000 y before the boundary near the base of Chron 29r, followed by a rapid return to cooler temperatures approximately 50,000 y before the boundary, which were then maintained through the earliest Paleogene [7],[20],[21]. This longer-term temperature change could have counteracted the hypothesized bolide-caused selection against traits that characterize “slow” strategies. Recent trait-climate theory suggests that cooling should lead to reduced evapotranspiration demand, directionally selecting for “slow” strategies (assuming no change in atmospheric [CO_2_]) [22],[23]. This prediction appears opposite to the predictions of Wolfe's deciduousness hypothesis, but the relative strengths of both predictions have been unknown.

We evaluate the causes and consequences of ecological change across the KPB by producing records of two central leaf functional traits that reflect variation in ecological strategies: leaf mass per area [LMA, g dry leaf mass/m^2^ leaf area (LA)], and leaf minor vein density (VD, mm vein/mm^2^ LA). Variation in these traits reflects a tradeoff between the transport of water and carbon [24] and the carbon construction cost of the leaf [23]. LMA is a metric of leaf carbon investment and correlates negatively with deciduousness and leaf lifespan [19],[25]. VD is a metric of the hydraulic and photosynthetic capacity of a leaf. Leaves with higher VD reflect “fast” species with higher rates of water flux and carbon assimilation [23],[24] and are more likely to be found in warm environments [22],[26]. Thus, by examining each trait's pre- and postboundary distribution, we can assess if the KPB extinction event was selective and which of the differing selection scenarios best matches temporal trends in leaf traits.

## Results

**Figure 1:**
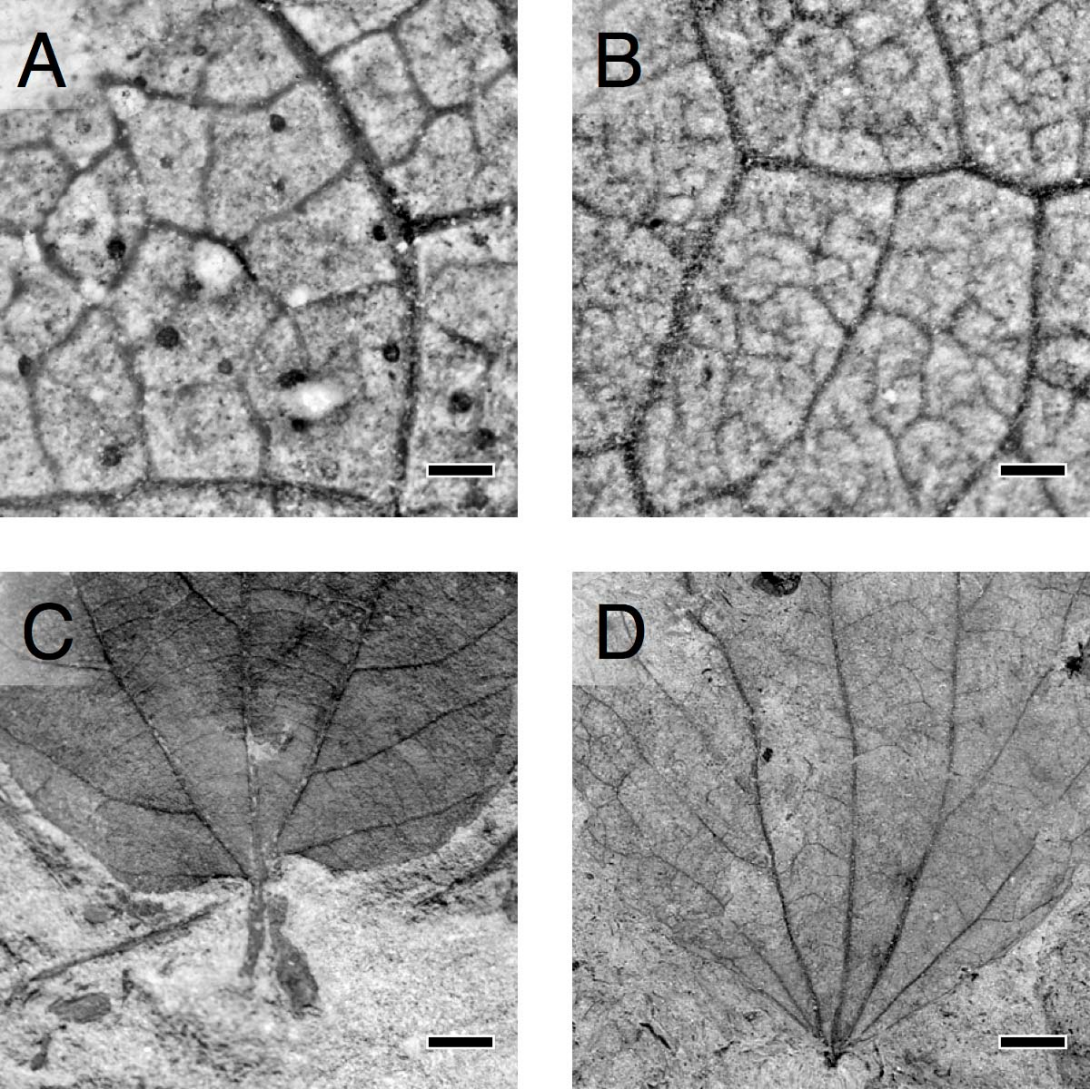
Visual representations of trait changes across the KPB. Top row, increase in VD as seen in (A), *“Dryophyllum” subfalcatum*, −30.7 m stratigraphic depth, VD = 2.5 mm^−1^ and (B) unknown nonmonocot (morphospecies FU87), 1.275 m depth, VD = 5.3 mm^−1^. Bottom row, decreases in LMA as seen through decreasing PW for similar LA in (C) *“Ficus” planicostata*, −3.6 m depth, LMA = 136 g m^−2^ and (D) *“Populus” nebrascensis*, 7.2 m depth, LMA = 48 g m^−2^. Scale bar, (A and B) 500 µm and (C and D), 5 mm.

We measured VD and LMA for leaf fossil assemblages spanning the last ca. 1.4 Myr of the Cretaceous (K) and the first ca. 0.8 Myr of the Paleogene (P) (Figure 1). Floras come from the Hell Creek and Fort Union formations in southwestern North Dakota, United States (paleolatitude 49°N) [27]. Of the known leaf macrofossil assemblages spanning the KPB, these are currently the best preserved and most species-rich.

**Figure 2:**
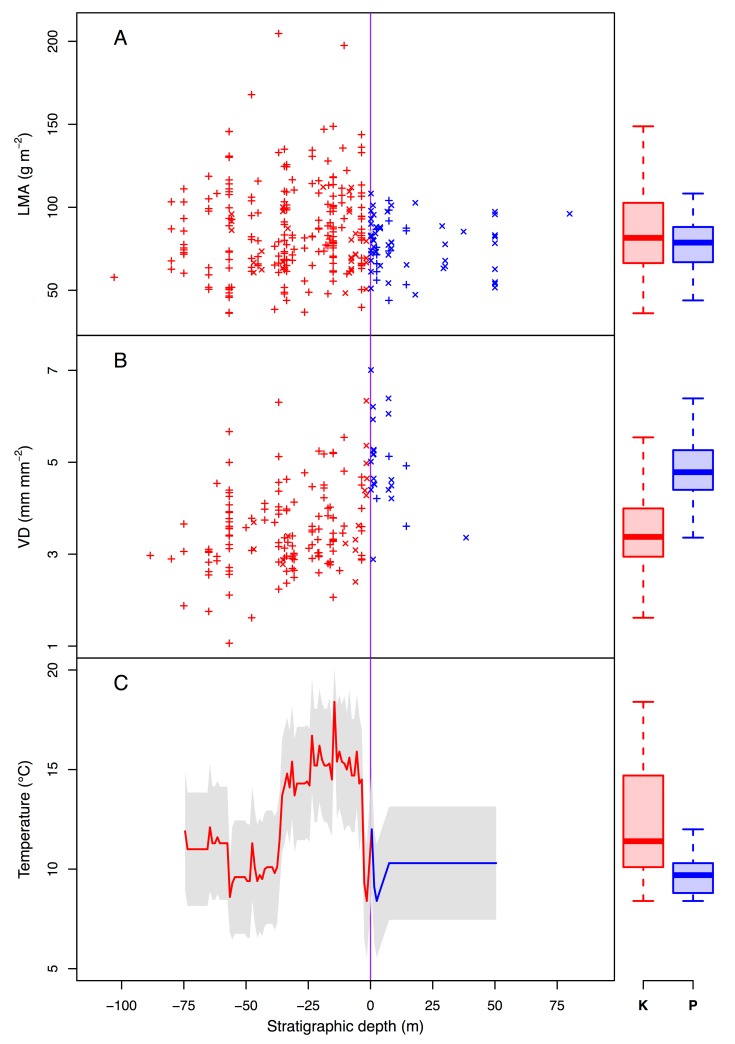
Changes in leaf functional traits and climate across the KPB (purple vertical line). (A) LMA reflects leaf carbon investment, with lower values associated with deciduous species. (B) VD reflects carbon assimilation capacity, with higher values associated with deciduous species. For (A) and (B), each symbol is a species at site mean: +, channel facies; x, floodplain facies. (C) Changes in temperature reconstructed previously from leaf-margin analysis of the same floras [20]. Color indicates stratigraphic depth, with red for Cretaceous data and blue for Paleogene data. Boxplots of each distribution are shown in the right margin.

**Figure 3:**
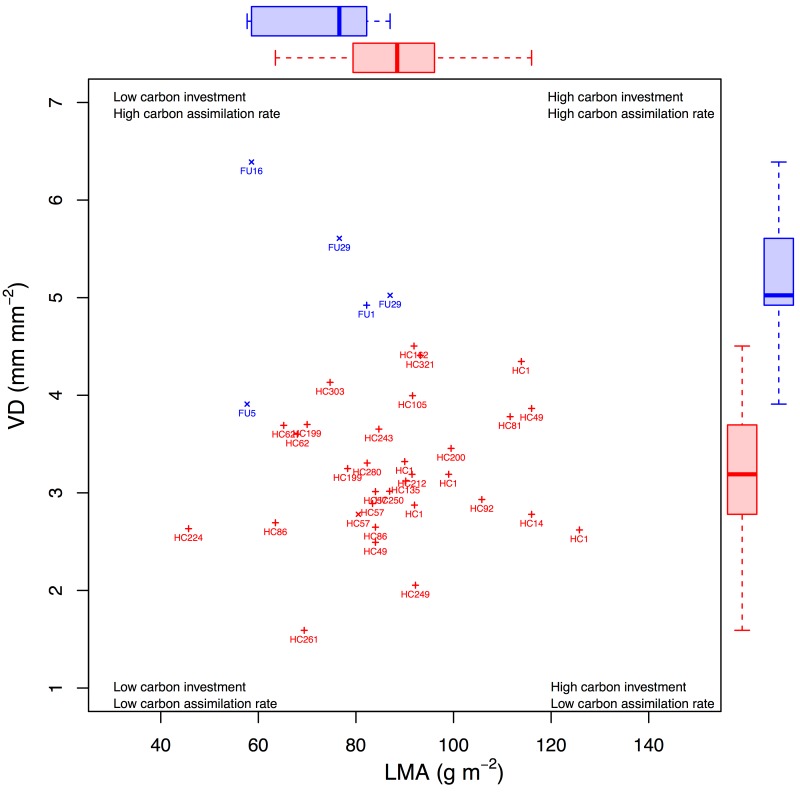
Ecological strategy dynamics across the KPB, as seen in a bivariate trait space of VD and LMA. Points represent individual specimens for which both VD and LMA measurements were available (labeled by morphospecies). Boxplots of each distribution are shown in the margins. Colors and symbols are as in Figure 2.

We found that the mean and variance in LMA decreased across the KPB. Mean values shifted from a Cretaceous value of 86±27 s.d. g m^−2^ to a Paleogene value of 78±15 s.d. g m^−2^ (Mann–Whitney location test, *p* = 0.04; Figures 2A and 3) [17]. This mean shift is small relative to the global range of LMA values (3–2,000 g m^−2^ [17]) but is of comparable magnitude to shifts across modern ecosystem types (e.g., from tropical rain forest to tropical deciduous forest, 73 versus 83 g m^−2^, respectively) [28]. Variance in LMA fell by 67% across the KPB (Brown–Forsythe Levene test for homogeneity of variances, *p*<10^−3^). These nonparametric tests account for unequal group sample sizes (*n_K_* = 256 and *n_P_* = 67).

We also found that the mean of VD increased across the KPB. Mean values shifted from a Cretaceous value of 3.5±0.6 s.d. to a Paleogene value of 4.6±0.7 s.d. mm^−1^ (*p*<10^−3^; Figures 2B and 3). This shift is comparable to the average difference between extant nonangiosperms and eudicots (1.8 versus 8.0 mm^−1^, respectively) [29]. Variance in VD did not change across the KPB (*p* = 0.66). Note that group sizes here were also unequal (*n_K_* = 146 and *n_P_* = 24).

The trends towards higher VD and reduced ranges of LMA reflect large physiological and biological shifts in plant functioning. The constriction of LMA space was caused primarily by the loss of high-LMA species, many of which were abundant in the latest Cretaceous (Figures 2A and S3). The shift in VD space was caused mostly by the loss of Cretaceous-only species with low VD relative both to species that survive the KPB and to Paleogene-only species (regression; *p*<10^−9^, *r* ^2^ = 0.22; Figure 2B).

Several of these patterns may be driven by sampling biases—for example, temporal variation in facies preservation [30]. In this dataset, Paleogene sites come primarily from channel facies, whereas Cretaceous sites usually come from floodplain facies. To assess the potential impact of differential preservation, we repeated analyses after subsetting by each facies type for each trait. For channel VD, the mean effect remained significant (*p* = 0.03; *n_K_* = 130 and *n_P_* = 4), as it did for floodplain VD (*p* = 0.004; *n_K_* = 16 and *n_P_* = 20). For channel LMA, there was no longer a significant shift in mean or variance (both *p*>0.11; *n_K_* = 222 and *n_P_* = 15). Similarly no effect in mean or variance was found for floodplain LMA (both *p*>0.29; n_K_ = 34 and *n_P_* = 52). Low Paleogene sample sizes and unavoidable facies shifts prevent further inferences. Nonetheless, together these results indicate a strong evidence for a shift in the mean of the VD distribution but yet weaker evidence for shift in the variance of the LMA distribution. Moreover, because channel (riparian) environments typically support more “fast-return” specialists than distal floodplain environments owing to their higher rates of physical disturbance and greater volatility in nutrient availability [31], our facies effect should have had the opposite effect on LMA and VD than what we observed.

Other data quality issues may also bias our results. First, estimates of VD may be underestimates of true values because of incomplete fossil preservation. Although this is an unavoidable problem when estimating VD for any leaf fossil, our protocol did exclude all but the best-preserved specimens (Materials and Methods). Reported values are consistent with other estimates from late-Cretaceous fossils [32]. Second, estimates of LMA may be down-biased if some species were incorrectly expert-determined to be herbaceous rather than woody. We therefore repeated all LMA analyses across and within facies, with herbaceous taxa removed (omitting *n* = 21 specimens). We found no qualitative changes in conclusions.

Shifts in both functional traits were associated with ongoing climate change. The bin-mean VD and temperature were negatively correlated (*r* ^2^ = 0.15, *p* = 0.01), whereas bin-mean LMA and temperature were positively correlated (*r* ^2^ = 0.19, *p* = 0.005). Similarly at the specimen level, VD and LMA were not correlated with each other across (*p* = 0.53) or within facies (both *p*>0.45), but this null result is likely due to the low number of available samples (channel, n_K_ = 1 and n_P_ = 4; floodplain, n_K_ = 31 and n_P_ = 1). Overall, these results indicate a systematic shift in trait space across the KPB whether examined at the bin-mean or specimen level.

## Discussion

The Chicxulub bolide impact appears to have led to the selective extinction of plant species with “slow” leaf ecological strategies. Consistent with Wolfe's hypothesis, this mass extinction was characterized by directional selection away from evergreen species [6], as seen through both VD and LMA, as well as stabilizing selection, as seen through LMA. Our study therefore provides strong evidence that the KPB mass extinction was functionally selective for plants.

The increase in VD from the late Cretaceous to the early Paleogene in our data parallels the increases in VD seen across angiosperm taxa in the early-mid Cretaceous and then again across the KPB [32]. The increase reported by Feild et al. (2011) occurred globally over an ∼70 Myr time span, with most of this increase occurring conclusively before the KPB. In contrast, our findings show a VD increase in a *single* region, over a much shorter time period (∼2 Myr), with the majority of the increase occurring at or after the KPB. Nevertheless, it is possible that similar atmospheric changes drove both trends. Higher VD in angiosperms and increasing angiosperm dominance across the Cretaceous could be driven by declining atmospheric [CO_2_], because lower carbon dioxide availability could select for higher hydraulic capacity to maintain productivity [33]. Trait-climate theory also predicts a negative correlation between temperature and VD under low [CO_2_] [22], consistent with observed data for the KPB. A hypothetical rapid [CO_2_] decrease across the KPB therefore could be a key driver to our findings of an observed shift in VD. One hypothetical driver of lower [CO_2_] could be enhanced chemical weathering, via late-Cretaceous Deccan volcanism [34]. However, extant KPB proxies do not yet have sufficient age control [35] or temporal resolution [35],[36] to accurately reconstruct finer scale [CO_2_] dynamics within the time interval of interest. More detailed assessments of atmospheric composition and temperature across the KPB would be needed before this prediction could be tested.

There is an important criticism of the proposed mechanism coupling between VD and [CO_2_]. Although selection against low hydraulic capacity in low [CO_2_] environments should occur for all plants, for nonangiosperms and shade species, low values of VD appear to have successfully persisted in the fossil record across the Cretaceous [37], suggesting that low values were not necessarily selected against as originally hypothesized [33]. We therefore suggest that the increase in VD seen across the KPB is more likely to be a direct consequence of the bolide impact selecting for specific leaf economic strategies rather than of ongoing longer-term climate change. Nevertheless, the increasing dominance of deciduous angiosperms by the early Paleogene appears to have been reinforced by both bolide impact and longer-term climate change.

Observed trait dynamics across the KPB likely ramify to influence ecosystem functioning. Because of the close linkage between leaf economic traits and ecosystem resource fluxes [9],[10], selection at the KPB should have also strongly modulated net primary productivity in terrestrial systems [28], as well as regional hydrological cycles [38]. Our results therefore suggest that there were associated functional changes in terrestrial ecosystems in the aftermath of the Chicxulub impact. Functional trait dynamics are of wide interest when studying succession, invasion, and other dynamical questions, but contemporary time-series data are very rare (e.g., [39]) Our study thus highlights the power of paleoecological functional trait data to integrate information on climate change, extinction, and species performance across time.

## Materials and Methods

### Data Sources

We analyzed fossils of nonaquatic nonmonocot angiosperm taxa from sites previously collected from the Hell Creek and Fort Union Formations, now located in southwestern North Dakota. The stratigraphy of these sites and the identification of specimens has been described previously [40]. Each specimen was previously assigned to one of 312 morphotypes and one of 208 sites corresponding to a known stratigraphic position and sedimentary facies.

### LMA Measurements

During the summer of 2013, we examined all appropriate specimens at the Denver Museum of Nature and Science (DMNH) and Yale Peabody Museum (YPM). We digitally photographed specimens with (1) intact petioles at the point of insertion with the leaf blade and (2) LAs that could be directly measured or confidently reconstructed. For each image, leaves were separated from their rock matrix using the lasso tool in Adobe Photoshop; LA and petiole width (PW) were then measured using ImageJ following the protocol of [41]. For woody species, LMA was then calculated using the following empirical scaling function [41]:
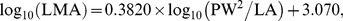
(1)and for herbaceous species [42]:
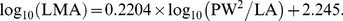
(2)The final dataset comprises 612 specimens representing 135 morphotypes and 102 sites, of which only 21 specimens are designated as herbaceous.

### VD Measurements

During June 2012, we examined all appropriate specimens at DMNH, including holomorphotypes on loan from YPM (approximately 6,000 specimens total). We selected specimens that appeared to have complete preservation of the minor venation network in at least one region of the fossil, discarding all others (retaining 1,150 specimens). In specimens containing more than one leaf, we made a measurement for each leaf. We captured a digital image of each leaf fossil centered on the region of interest using a dissecting microscope coupled to a digital camera (T2i, Canon). Illumination was provided by ring-light. Image resolution was 243 pixels per millimeter.

We then estimated VD using a line-counting program [22] developed in MATLAB. To prevent any investigator bias, images were unlabeled and analyzed in random order. We converted each image to grayscale and applied a contrast limited adaptive histogram equalization to improve quality. If vein preservation appeared incomplete (e.g., clear fragmentation of specimen, higher-order veins preserved in one region but not in another, dramatically fewer veins in one of two conspecific specimens), we discarded the specimen at this step. For acceptable specimens, we delineated a polygonal region of interest in each image (corresponding to the area in which veins were potentially preserved). The program then generated a randomly oriented line segment spanning this polygon. We manually counted the number of vein-line intersections and repeated this process for 10 random line segments. We then computed the mean distance between veins as the sum of all line counts divided by the sum of all distances. This distance was then converted to VD via an equation [22] stating that VD (mm^−1^) and intervein distance (d, mm) are related as:
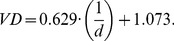
(3)This procedure yielded a final dataset of 468 specimens from 66 species and 61 sites.

### Facies Assignment

Each specimen was originally assigned a facies type by the original collector (table 3 in [40]). Channel facies were defined as those recorded as “aban chan,” “channel,” “channel la,” “channel x,” or “Colgate channel.” Floodplain facies were defined as those originally recorded as “ash bed,” “carb shale,” “carb splay,” “floodplain soil,” “pond,” “pond ls,” “pond vb,” “splay,” “splay/levee,” or “volcanic ash.”

### Stratigraphic Binning

We calculated time series of VD and LMA using a binning approach. We first assigned each specimen to a 1-m depth interval defined by integer-rounding the measured stratigraphic depth. Within each bin we calculated species-at-site mean trait values, then used these to compute site means. We used these site-means to infer the distribution (i.e., mean, upper quartile, lower quartile) of trait values within each bin. Because VD and LMA data were not always available within the same bin, we used piecewise linear interpolation to infer LMA values at each bin for which VD values were available (*n* = 40).

### Paleo-Temperature Data

An index of temperature (T_c_; °C) was obtained from a leaf margin analysis of the same fossil floras using the range-through mean annual temperature values corresponding to the supplementary table 8 of [20]. These leaf-reconstructed temperatures are supported by carbonate clumped isotope paleothermometry from the same region [21]. Because vein data and margin data were not always available for the same stratigraphic bins, we estimated temperatures with a piecewise linear interpolation of range-through temperature against stratigraphic bin, choosing constant values at endpoints. We modeled the uncertainty in T_c_ using the error estimates provided in the original source, using the mean plus (minus) one standard deviation as the upper (lower) quartile. When no standard deviations were available, we assigned a standard deviation equivalent to the mean of all other standard deviations (2.8°C).

## Supporting Information

Figure S1 VD data, broken out by stratigraphy and facies. Symbols indicate species at-site means. Black points, data for each category; gray points, all other data.
(PDF)

Figure S2 LMA data, broken out by stratigraphy and facies. Symbols indicate species at-site means. Black points, data for each category; gray points, all other data.
(PDF)

Figure S3 LMA of species with different stratigraphic ranges and abundances. High LMA leaves are common in species that are found only in the Cretaceous but drop out at all abundances in species that cross the KPB or are found only in the Paleogene. Each point represents a species. Abundance data come from the quantitative census of Wilf and Johnson [4].
(PDF)

Figure S4 Correlations between traits for time series data shown in Figure 2. Data for VD and LMA have been aggregated into 1 m bin grand means (across species at-site means and across sites). Points are colored as in Figure 2.
(PDF)

Data S1 VD data for all measured specimens.
(CSV)

Data S2 LMA data for all measured specimens.
(CSV)

Data S3 Temporally binned VD and LMA values, paired with proxy temperature values.
(CSV)

## Abbreviations

K: Cretaceous
KBP: Cretaceous–Paleogene boundary
LA: leaf area
LMA: leaf mass per unit area
P: Paleogene
PW: petiole width
VD: vein density
