# Supplementary figures and images for: Plant Ecological Strategies Shift Across the Cretaceous–Paleogene Boundary
Source: PLoS Biol. 2014 Sep 16;12(9):e1001949. doi: 10.1371/journal.pbio.1001949 (PMC4165584; doi:10.1371/journal.pbio.1001949)

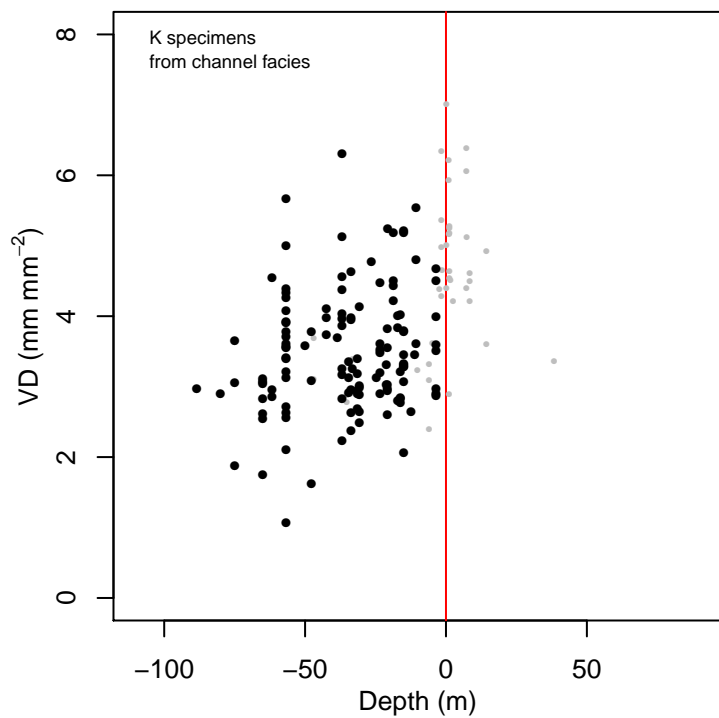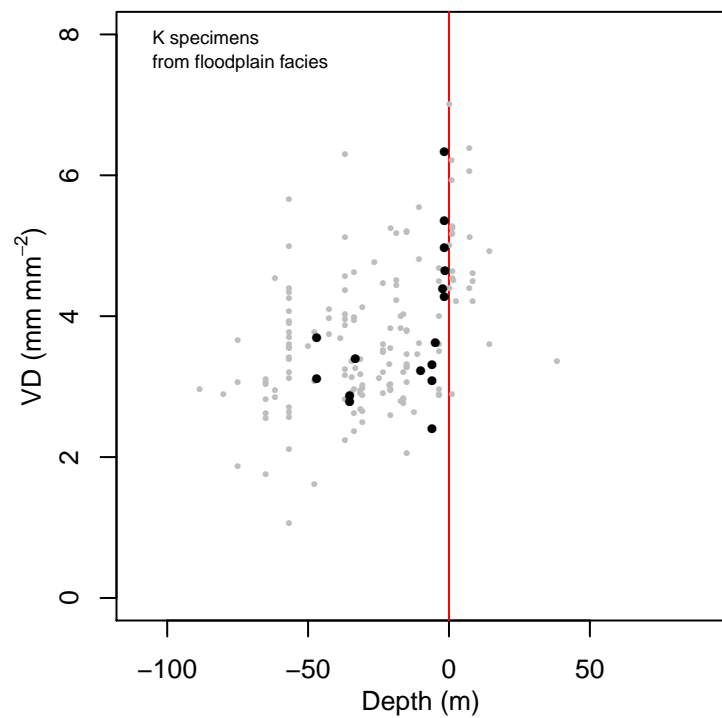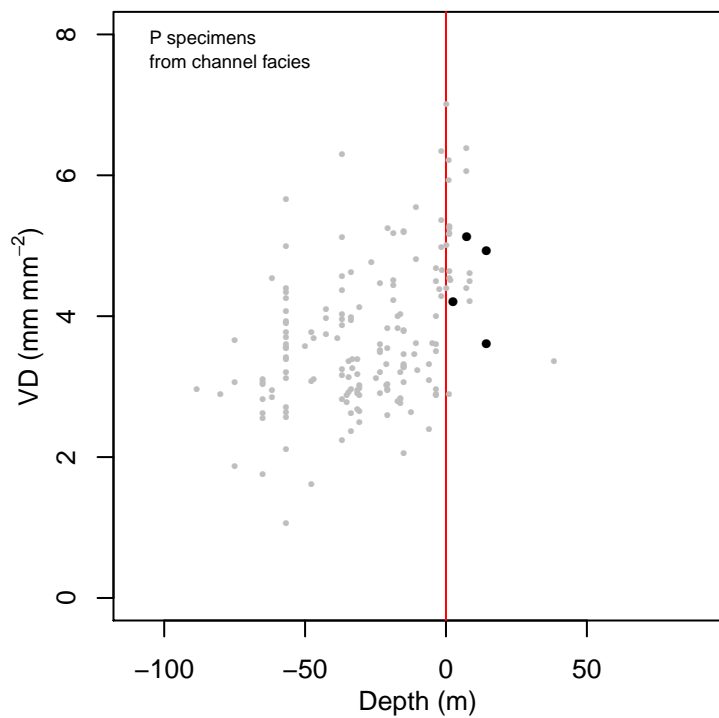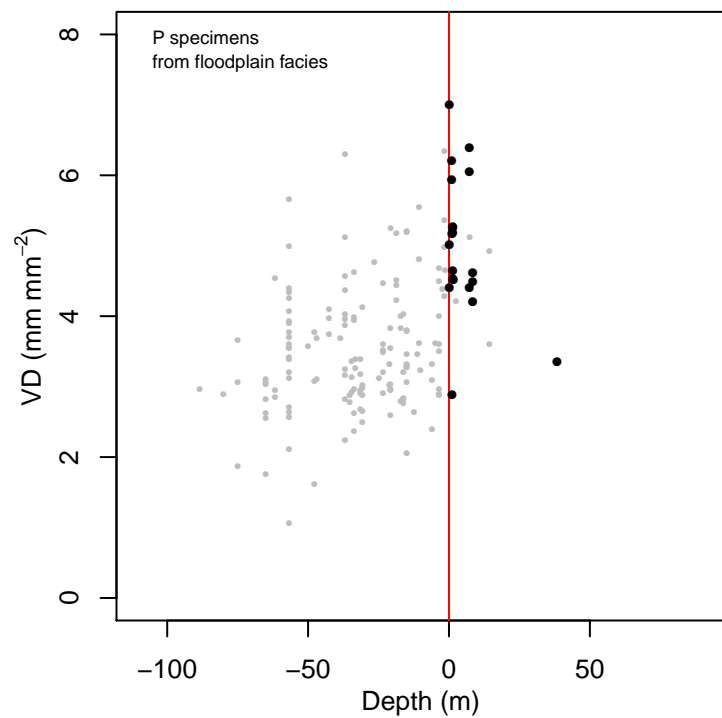

Supplement: Figure S1 — VD data, broken out by stratigraphy and facies. Symbols indicate species at-site means. Black points, data for each category; gray points, all other data. (PDF) [file pbio.1001949.s001.pdf]

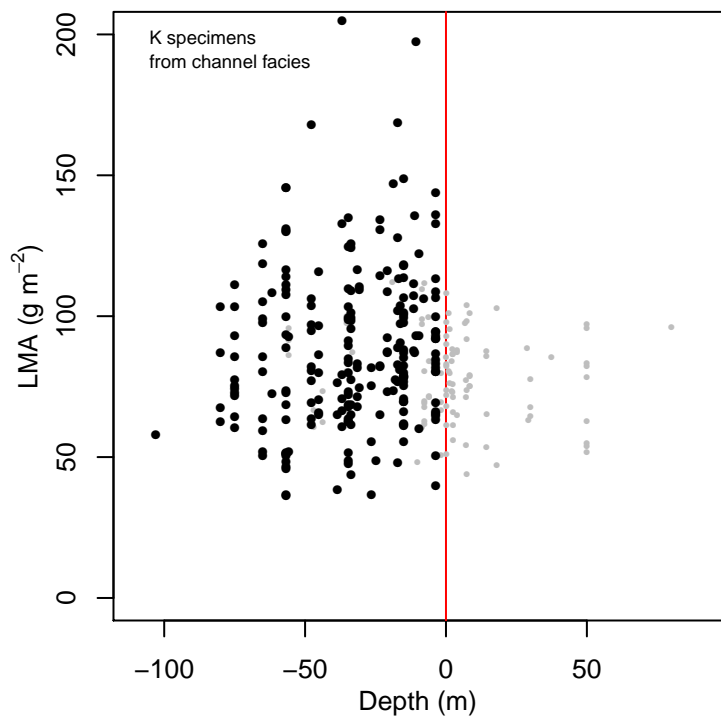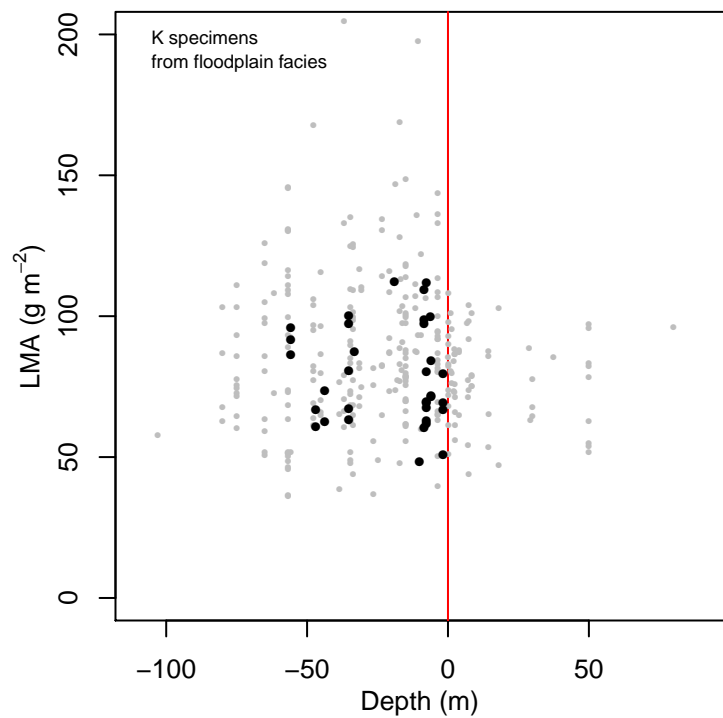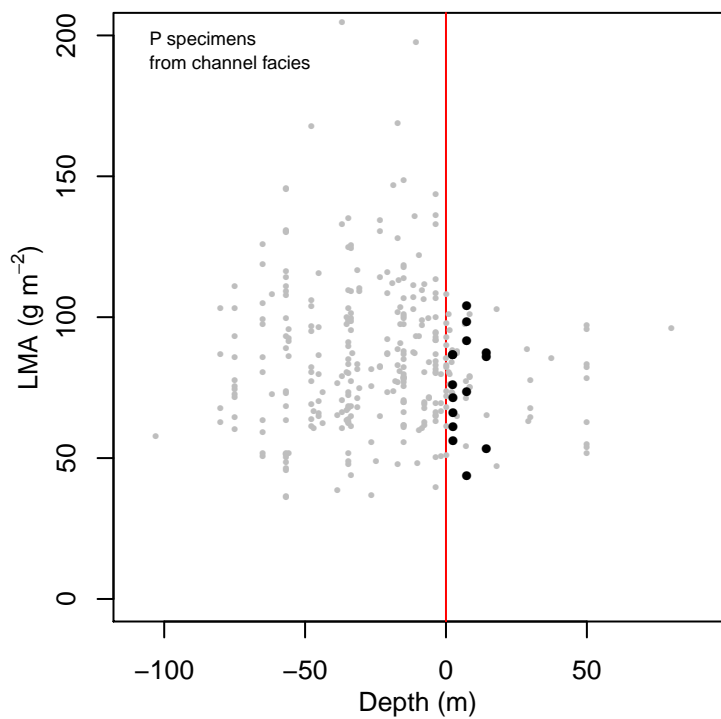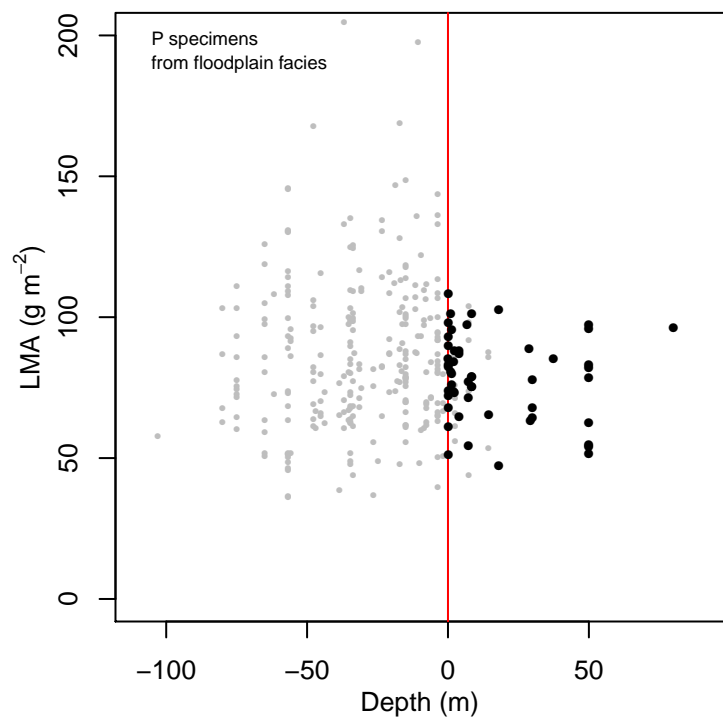

Supplement: Figure S2 — LMA data, broken out by stratigraphy and facies. Symbols indicate species at-site means. Black points, data for each category; gray points, all other data. (PDF) [file pbio.1001949.s002.pdf]

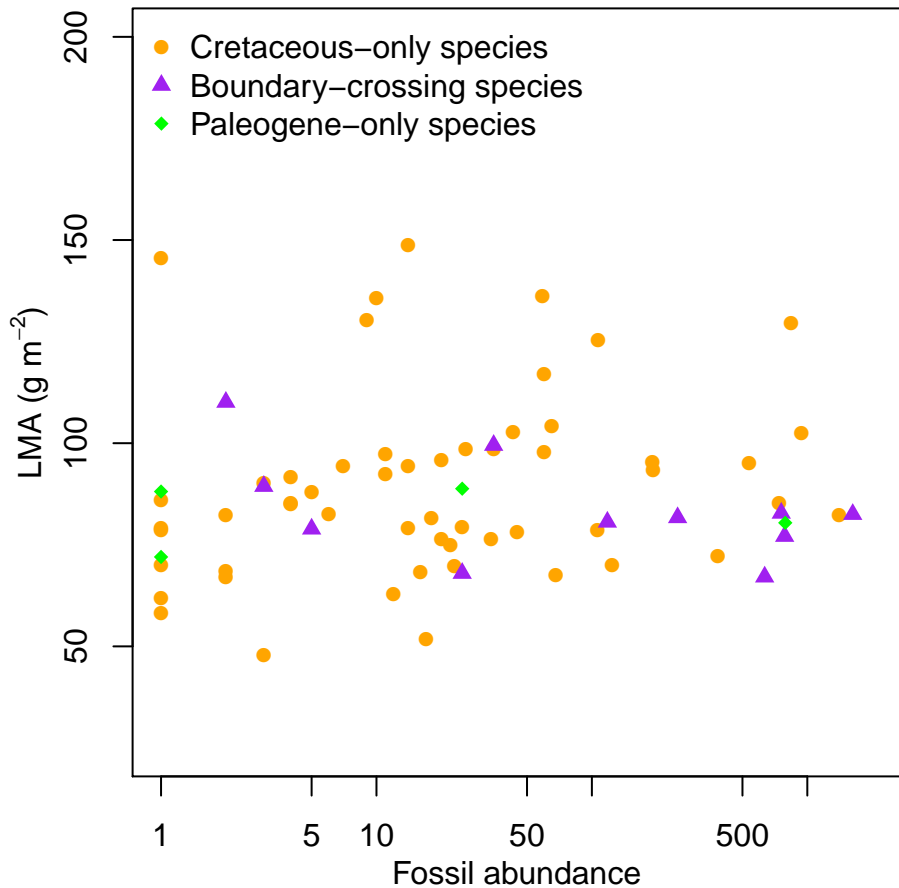

Supplement: Figure S3 — LMA of species with different stratigraphic ranges and abundances. High LMA leaves are common in species that are found only in the Cretaceous but drop out at all abundances in species that cross the KPB or are found only in the Paleogene. Each point represents a species. Abundance data come from the quantitative census of Wilf and Johnson [4]. (PDF) [file pbio.1001949.s003.pdf]

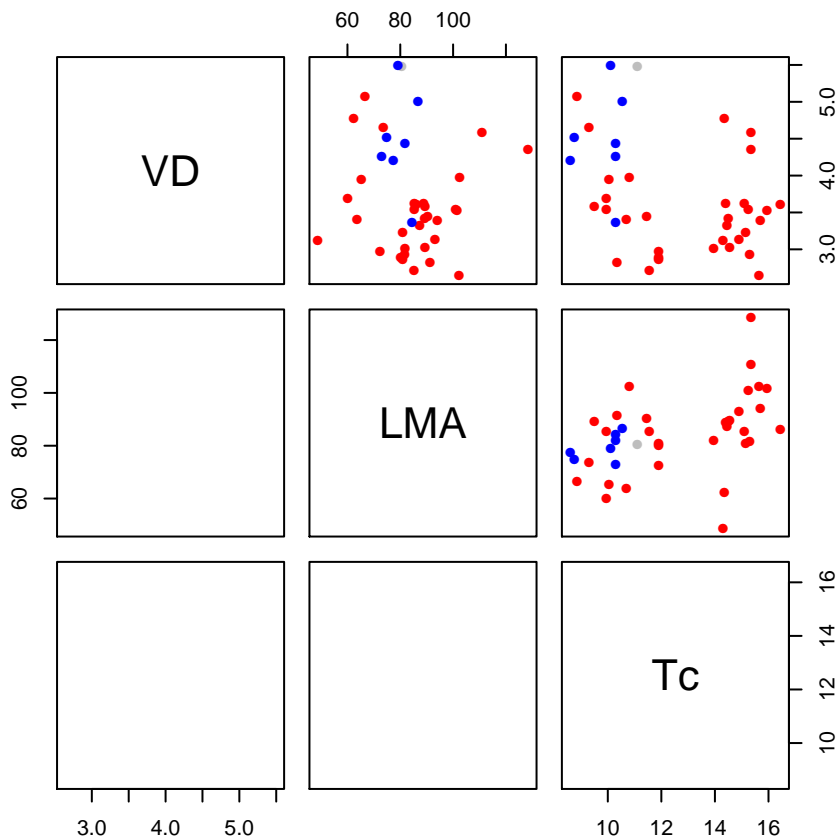

Supplement: Figure S4 — Correlations between traits for time series data shown in Figure 2. Data for VD and LMA have been aggregated into 1 m bin grand means (across species at-site means and across sites). Points are colored as in Figure 2. (PDF) [file pbio.1001949.s004.pdf]
